# 25-OH Vitamin D and Interleukin-8: Emerging Biomarkers in Cutaneous Melanoma Development and Progression

**DOI:** 10.1155/2015/904876

**Published:** 2015-10-04

**Authors:** Corina-Daniela Ene, Amalia-Elena Anghel, Monica Neagu, Ilinca Nicolae

**Affiliations:** ^1^Nephrology Hospital “Dr. Carol Davila”, 4 Calea Grivitei, 010731 Bucharest, Romania; ^2^SKINMED Clinic, 8 Splaiul Unirii, 040102 Bucharest, Romania; ^3^Immunology Department, Victor Babes National Institute of Pathology, 99-101 Splaiul Independentei, 050096 Bucharest, Romania; ^4^Faculty of Biology, University of Bucharest, 91-95 Splaiul Independenţei, 76201 Bucharest, Romania; ^5^Department in Dermatology, Infectious and Tropical Diseases “Dr. Victor Babeş" Clinical Hospital-Research, 281 Soseaua Mihai Bravu, 030303 Bucharest, Romania

## Abstract

*Background*. There are several circulatory biomarkers that are involved in forecasting the clinical outcome of cutaneous melanoma. Serum/plasma vitamin D status is one of the markers intensively studied in this type of cutaneous cancer. The combination of validated serum biomarkers (like LDH) with new biomarkers such as IL-8, angiogenic factor, and vitamin D is still at the dawn of research. Hence, we are aiming to establish the predictive power of inflammatory biomarkers, such as IL-8, and metabolic ones, such as vitamin D. These candidate biomarkers are intended to aid classical biomarkers, such as LDH, in the prognosis of cutaneous melanoma.* Methods.* Serum vitamin D and IL-8 were quantified in melanoma patients and in matching healthy controls.* Results.* Median serum vitamin D concentrations were significantly lower (*p* = 0.003) in melanoma patients as compared to healthy control subjects, while around 65% of the investigated patients have proven a severe circulatory deficiency of this vitamin. IL-8 was found increased (*p* = 0.001) in melanoma patients as compared to controls.* Conclusion.* Upregulation of proangiogenic factors associated with vitamin D deficiency can prove to be potent future biomarkers candidates, enhancing the predictive power of classical LDH.

## 1. Introduction

A great amount of scientific information has been gathered regarding “vitamin D/cancer hypothesis” [1]. Sun radiation is an environmental factor that can trigger cutaneous melanoma on the one hand, while sun exposure induces skin-mediated synthesis of vitamin D3, on the other hand. There are plentiful pro- and retrospective epidemiological studies showing that low serum 25-hydroxyvitamin D3 (25(OH)D) concentrations can be associated with colorectal [2], lung [3], and breast [4] cancer and with various other types of cancer [5]. This association was related with a significantly increased risk for the worst development of the disease and an overall poor prognosis [6]. Besides these studies, there are also several reports showing high serum 25(OH)D association with lower cancer incidence [7].

Taking into account all the gathered information regarding cutaneous melanoma development, we have embarked in studying the basal serum levels of 25(OH)D in patients diagnosed with cutaneous melanoma. Knowing that there is no “perfect multipotent biomarker,” we have been studying the 25(OH)D serum level in correlation with classical validated ones, like LDH and newer candidates, like the proangiogenic cytokine IL-8.

*Choosing Future Biomarkers.* There are several reasons for which we have chosen the presented parameters in melanoma patients and the reasons are highlighted herein.

Interleukin-8 (IL-8), described almost 20 years ago, is a cytokine produced by immune cells (monocytes, macrophages) as well as nonimmune cells (endothelial cells, keratinocytes, melanocytes, and chondrocytes) and tumor cells [8]. IL-8 has a strong capacity to specifically activate neutrophils, inducing intracellular calcium rise, reactive oxygen generation, chemotaxis, and enhancement of adhesion molecules expression. Keratinocytes respond to TNF-alpha's action with an IL-8 increased synthesis. IL-8 secretion is correlated with the increased secretion of other chemokines like MCP-1 and IP-10 through NFkappaB activation [9].

IL-8 is produced by several tumor cells and hence its functions include regulation of tumor angiogenesis and immune response. IL-8, along with other chemokines, like CXCL-1 produced by melanoma, has strong influence on primary tumors progression [10] and is able to significantly stimulate Keratin 8 expression in cultured keratinocytes [11].

Overexpression of IL-8 by neoplastic cells seems to be regulated by the hypoxia generated by growing tumors. IL-8 overexpression increases CXCR1 and CXCR2 expression on tumor cells, endothelial cells, and immune related cells (neutrophils and macrophages) resident in tumor tissue [12].

Earlier studies have shown that, in melanoma cell lines, 1-alpha, 25-Dihydroxyvitamin D3 (calcitriol) repressed IL-8 promoter activity that was induced by tumor necrosis factor-alpha (TNF-alpha). This effect was consistent with the one developed by TNF-alpha upon IL-8 release and upon IL-8 mRNA levels. However, these early studies have shown that only vitamin D3 metabolites, which were able to transactivate a classical vitamin D response element, had the ability to repress IL-8 promoter activation. This finding suggested that the repression is mediated* via* vitamin D receptor (VDR) [13].

Further on, it has been shown that 20-hydroxyvitamin D(2) [20(OH)D(2)] inhibits DNA synthesis in epidermal keratinocytes and in melanoma cells. Transformed melanocytes are more sensitive than normal melanocytes. A noncalcemic analog of vitamin D, 20(OH)D(2), proves cell-type dependent antiproliferative and prodifferentiation activities through activation of VDR, with no detectable toxic calcemic activity [14]. Subsequent studies have shown that indeed melanoma cells express VDR and new insights into the molecular mechanisms that underlie 1,25(OH)(2)D(3)-sensitivity in melanoma cells were published. Melanoma cells responsiveness to 1,25(OH)(2)D(3) corresponds to the expression level of VDR mRNA, which in turn might be regulated by VDR microRNAs or epigenetic modulating drugs [15]. Recently, in a large German patients cohort diagnosed with cutaneous melanoma, it was highlighted that a decreased 25(OH)D level is associated with increased tumor thickness and advanced tumor stage [16].

Lactate-dehydrogenase (LDH) is a validated serum biomarker that evaluates the metastasis risk [17–20] and it actually evaluates the tissue leakage induced by tumor growth and metastasis. An association of the LDH tissue leakage induced by skin damage with an increase of mRNA IL-8 level was reported [21].

In the light of the updated information and in the quest to enhance the panel of biomarkers that could diagnose and prognosticate the cutaneous melanoma outcome, we have investigated the 25-(OH)D serum concentrations of untreated melanoma patients in correlation with classical serum biomarkers like LDH and possible new biomarkers like IL-8.

## 2. Materials and Methods

### 2.1. Patients

88 patients (57 women and 31 men) diagnosed with cutaneous melanoma according to AJCC [22] were monitored for 2 years. The presented results were obtained after diagnosis and no installed therapy. Diagnostic was obtained after applying AJCC criteria. The enrolled patients were as follows: Clark II (19.3%), Clark III (37.5%), Clark IV (25%), and Clark V (18.2%). The prevalence of women versus men was prior highlighted by us [23] where consecutive enrollment of melanoma patients has shown a mean of 75% women in the entire enrolled group as compared to 80% women in this study. In EU, this figures show also a prevalence of cutaneous melanoma in women [24].

### 2.2. Healthy Subjects

Healthy subjects consisted of an equal number of subjects (55 women and 33 men) and are selected from the same geographical regions as the patients (Bucureşti, Argeş, Dîmbovița, Giurgiu, Călăraşi, Constanța, Prahova, Ialomița, and Teleorman).

Patients and healthy subjects have provided their written informed consent. The study and consent procedure were approved by the involved Hospital's Ethics Committee. All the named institutional ethics committees specifically approved this study (ethics committees of Nephrology Hospital “Dr. Carol Davila,” Bucharest, Romania; of SKINMED CLINIC, Bucharest, Romania; of “Victor Babes” National Institute of Pathology, of Infectious and Tropical Diseases “Dr. Victor Babeş”).

The inclusion and exclusion criteria for the presented study are depicted in Table 1.

### 2.3. Biological Samples

Blood was harvested from patients and healthy subjects and serum was separated upon standard conditions [25].

*Serum Quantification of 25-OH Vitamin D.* Quantification was performed with ELISA using Euroimmun kit [26]. The technique has a 1.6 ng/mL sensitivity for 25-OH vitamins D2 and D3 and no cross-reaction with 24,25(OH)_2_ vitamin D3, vitamin D2 (ergocalciferol), and vitamin D3 (cholecalciferol). Briefly, anti-25-OH vitamin D3 antibodies detect the presence of 25-OH vitamin D3 indicated by peroxidase activity, measured as substrate OD at 450 nm. Results are expressed as ng/mL or nmol/L, after the following formula:* 25-OH vitamin D3 (ng/mL) × 2.5 = 25-OH vitamin D3 (nmol/L)*.

### 2.4. Serum Quantification of IL-8

Quantification was performed with ELISA using Enzo Life Sciences kit, with a detection limit of 0.64 pg/mL [27].

### 2.5. Serum Lactate-Dehydrogenase

(LDH) was quantified using standard photometric method (Human Diagnostics) and the results were expressed in U L^−1^.

### 2.6. Standard Laboratory Evaluation

Hematology and general biochemistry were evaluated in an Automated Biochemical Analyzer.

### 2.7. Statistics

SPSS program was used for data analysis; comparison was performed using Student's *t*-test for *p* < 0.05. For several comparisons, the ANOVA multiparametric test was used. The correlation was established using linear regression and Pearson correlation. The results are presented as mean with standard deviation and as prevalence, the proportion of normal subjects/patients with a specific parameter.

## 3. Results

Overall biochemical parameters of the investigated patients are presented in Table 2. As seen, there were no statistically significant differences between lipids, triglyceride, bilirubin, calcemia, phosphatemia, alkaline phosphatase, iPTH, CRP, and calciuria in patients compared to controls.

**Figure 1:**
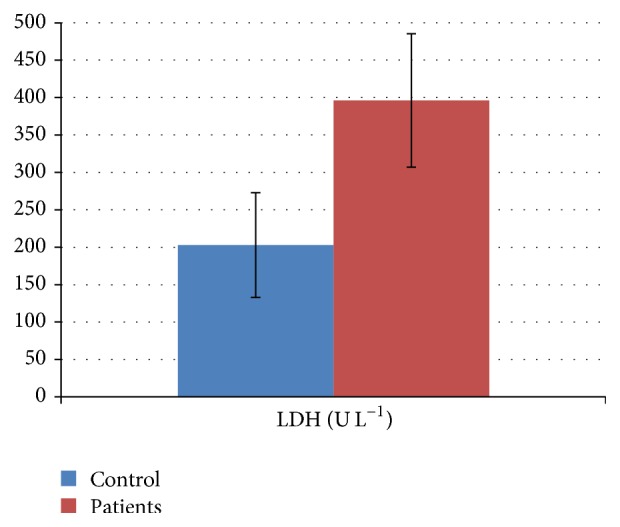
Serum LDH (U L^−1^) in cutaneous melanoma patients compared to healthy controls. Statistically significant differences were registered for serum LDH (*p* = 0.014).

**Figure 2:**
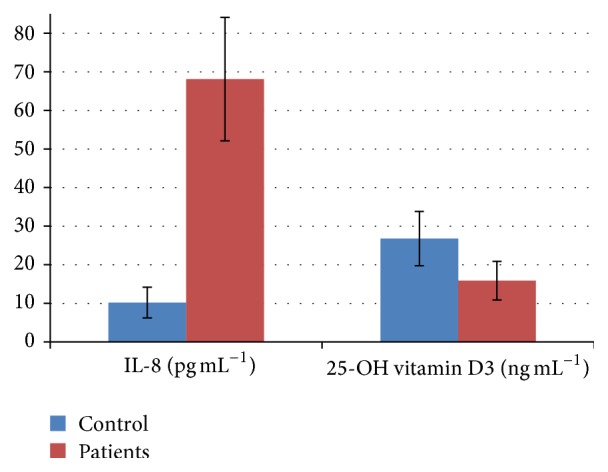
Serum IL-8 (pg mL^−1^) and 25-OH vitamin D3 (ng mL^−1^) in cutaneous melanoma patients compared to healthy controls. Statistically significant differences were registered for serum IL-8 (*p* = 0.001) and for 25-OH vitamin D3 (*p* = 0.003).

While standard biochemical parameters are not changed in the melanoma group, serum LDH, IL-8 and vitamin D3 are statistically significantly different (Figures 1 and 2).

Control IL-8 serum values match our previously published report [23], while patient values are elevated in comparison to our previous report. These differences reside first in the different patients' group population and secondly in the different used methods, ELISA versus multiplex technology. Patients' values for serum IL-8 are matching the overall range previously published when using ELISA as detection method [28].

Serum LDH is statistically increased in patients group compared to controls. The general accepted reference level for serum LDH is in the range of 135–450 U L^−1^. In 63.63% from the investigated cases, the registered value was in the reference level, while in the other 36.37% cases it was higher than the reference levels. For controls, 97.73% proved serum LDH values in the reference domain.

Regarding serum IL-8 concentration, 84.1% from the investigated cases had high values, while only 15.9% had values in the reference domain. In controls, 99% of the donors had lower than 15.0 pg mL^−1^ serum concentration of IL-8.

The serum level for 25-OH vitamin D3 is highly influenced by age, sex, diet, geographical location of the individual, sun exposure, and so on. There is still no consensus regarding the so-called normal value range for serum 25-OH vitamin D3. We have chosen healthy individuals from various geographical regions in Romania, both southern regions and lower mountain regions. Healthy donors were chosen to match the same geo/demographical and dietary lifestyle as the patients. Hence, the normal values that we present herein are the optimum normal values for the investigated patients group in the 18–45 years of age range, without claiming that it is the optimal range for the overall Romanian population.

As seen in Figure 2 and Table 3, patients have a clear vitamin D3 deficiency in comparison to controls, a mean of almost 50% reduction compared to healthy individuals. More than 50% of the normal donors have low levels of 25-OH vitamin D3 (less than 30 ng mL^−1^, Table 3).

**Figure 3:**
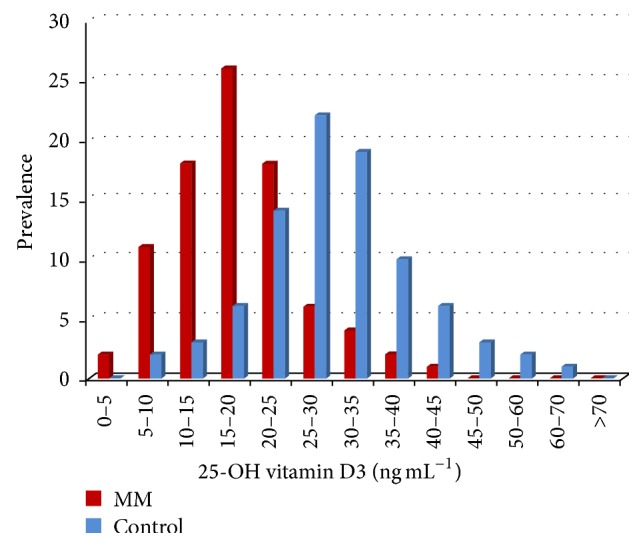
25-OH vitamin D3 serum level (ng mL^−1^) in melanoma patients (MM) compared to controls. Prevalence depicts the subgroups of low serum 25-OH vitamin D3 in the melanoma group of patients.

**Figure 4:**
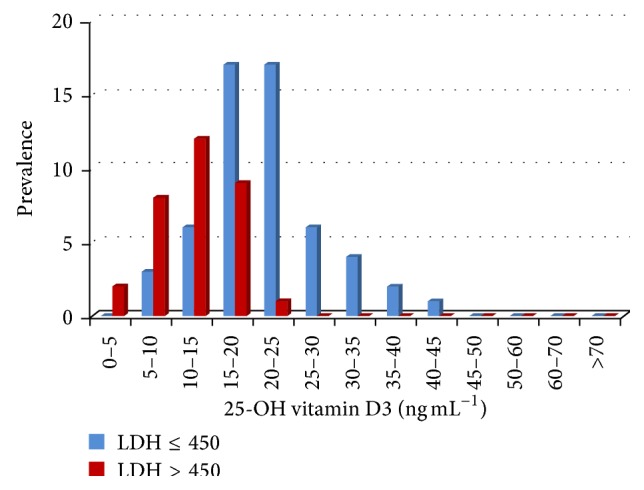
25-OH vitamin D3 (ng mL^−1^) serum level in melanoma patients for individuals with normal and high LDH serum levels (U L^−1^). Prevalence depicts the subgroups of melanoma patients with high serum LDH congregating in the low serum 25-OH vitamin D3 ranges.

**Figure 5:**
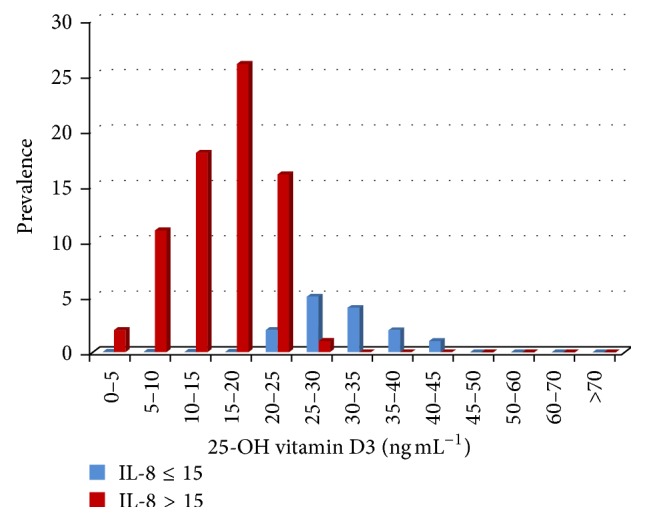
25-OH vitamin D3 (ng mL^−1^) serum level in melanoma patients for individuals with normal and high serum IL-8 (pg mL^−1^). Prevalence depicts the subgroups of melanoma patients with high serum IL-8 congregating in the low serum 25-OH vitamin D3 ranges.

From the data gathered and presented in Table 3 we have generated Figures 3, 4, and 5 depicting the subgroups displaying low, medium, normal, and high concentration of serum vitamin D3. Vitamin D3 deficiency has a higher prevalence in melanoma patients; only 8.0% display an optimum level (range 30–50 ng mL^−1^), while 92.0% have low serum levels (Figure 3).

The identified deficiency is correlated with modifications of both serums LDH and IL-8 in melanoma patients. Therefore, there is a negative correlation between serum 25-OH vitamin D3 and IL-8 (*r* = −0.650, *p* = 0.005) and LDH (*r* = −0.426, *p* = 0.021). In controls, no correlations between these parameters were identified. Clinically, serum 25-OH vitamin D3 deficit associated with high serum LDH and IL-8 indicated a metastatic risk for the investigated patients (Figures 4 and 5).

When evaluating the statistics and using linear regression, we have obtained a clear positive correlation between serum LDH (the only validated serum biomarker in cutaneous melanoma) and the circulatory levels of IL-8, a protumoral and proangiogenic factor (Table 4). Negative correlation was obtained between serum vitamin D3 and IL-8 and/or LDH (Table 4).

As studies over biomarkers discovery abound in melanoma, there is a continuous need of new serum biomarkers pin-pointing melanoma progression [29]. Our study stresses the fact that LDH, as a single approved marker, cannot spot the complex disease's evolution. Combining this classical biomarker with vitamin D3 and IL-8 can increase the predictive power of biomarkers and provide efficient rationale for follow-up and various treatment choices.

## 4. Discussion

The search for new prediction biomarkers in cutaneous melanoma, early detection, and prognosticators is still an unmet need. We have shown herein that melanoma progression is associated with a decrease in the serum level of 25-OH vitamin D3. There is a clear deficit of this vitamin in patients compared to controls, and this deficiency is negatively correlated with conventional biomarkers, such as LDH. When transition from the horizontal to vertical growth starts in cutaneous melanoma, and the metastatic process initiation is triggered, there is a drastic decrease of serum 25-OH vitamin D3, while LDH (tissue destruction) and IL-8 (angiogenesis) increase.

A decrease of 25-OH vitamin D3 serum level represents a bad clinical outcome of melanoma. These results join the confirmation studies that are conveyed towards vitamin D3 involvement in tumorigenesis, invasion, and metastasis.

There are still contradictions related to vitamin D3 and melanoma development. While several studies have shown positive association between dietary vitamin D3 intake and melanoma incidence [30], others have flagged a negative correlation [7] or the lack of any correlation [31].

Knowing that UV radiation is an environmental risk factor for cutaneous melanoma, but a trigger for vitamin D synthesis, we have enrolled patients and controls from geographical regions with higher and lower sun radiance, keeping the same UV index. In terms of environmental exposure, we did not find any correlation between the geographical region and vitamin D serum level.

The circulatory levels of vitamin D that we report in controls match the previously reported range in German population. The vitamin D data for patients presented herein are below the ones published for the German melanoma patients. In the German population, previous reports showed that the decreased vitamin D serum levels were associated with increased tumor thickness and advanced tumor stage [16]. In recent research studies, IL-8, an intense studied proangiogenic factor, was reported as one of the cytokines secreted by melanoma cells, influencing thus the differentiation pattern of keratinocytes and being a factor that mediates intercellular interactions in melanoma [32]. Moreover, it was shown that elevated IL-8 secretion and change in CXCR expression enhance melanoma malignancy [33]. Results performed in several solid cancers showed that serum IL-8 indicates tumor burden and prognosis in melanoma as well [34]. In our study, besides the correlation with the presence of melanoma tumor, the elevated serum IL-8 statistically correlated with an enhanced LDH and a reduced circulatory vitamin D.

We can speculate an intricate regulatory mechanism portrayed by these biomarkers. Thus, knowing that vitamin D metabolites have a repressive effect on IL-8 promoter activation [13] we can ascertain that low levels of vitamin D can turn off vitamin D receptor (VDR) stimulation, lowering IL-8 gene repression and hence an increase of proinflammatory IL-8 expression. Inflammatory milieu as proven in many types of cancer (here represented by IL-8) go hand in hand with an active tumor cell metabolism, represented by high levels of LDH [35]. Other inflammatory markers, as CRP and galectin-3, were prior correlated in melanoma with an increase in LDH [36] and circulatory markers such as S100 and MIA [37]. Recently it was demonstrated in normal melanocytes that triggering the apoptosis and hence the LDH release, genes encoding for proinflammatory cytokines like IL-8, are activated as well [38].

In the light of the presented results, we can draw a preliminary biomarker pattern for enhanced risk of developing the metastasis stage in melanoma for patients that have vitamin D deficiency, doubled by an increased circulatory IL-8 and LDH.

## 5. Conclusions

The direct relation between sun exposure and melanocyte malignant transformation is complex. Upon sun exposure, melanoma risks increases, especially in the case of sunburn, when inflammation occurs [39]. If vitamin D3 levels are decreased, unbalanced metabolic disorder can lead to further uncontrolled tumorigenesis. On national level, there is no large cohort screening for vitamin D3 level; thus we were confronted with the lack of information regarding the normal values and the population deficit.

Therapeutically, using vitamin D3 or its analogs can induce toxic effects (immune-suppression, hypercalcemia, multiple organ insufficiency, and death), but the recently developed new analogs have less toxicity and proved antitumoral effects in* in vitro* studies [40]. Low levels of circulatory vitamin D3 are much more frequent in melanoma patients compared to controls (92% versus 53%); these levels could indicate a high risk patient subpopulation and, using the new possibilities to therapeutically correct this deficiency, assist melanoma patients management.

Although imaging new technologies is evolving in dermatooncology [41], finding new biomarkers and/or enlarging the biomarkers panel for monitoring melanoma patients with parameters as vitamin D and IL-8 could both aid the prognosis of the disease and could identify high risk subgroups.

## Figures and Tables

**Table:** (a) Patients

Inclusion criteria	Age	18–45 years
	Sun exposure	UV index 5
	Nutrition, body mass index	Normal, 18.5–24.9
	Stage	Clark II, Clark III, Clark IV, and Clark V

Exclusion criteria	Age	>18 years; <45 years
	Pregnancy	Yes
	Condition present	Drug and/or alcoholic addiction
	Treatment present	Systemic treatment with hormones, antidepressants, antioxidants, and immunomodulators
	Other pathologies	Neurological, physiatrical, digestive, endocrinological, and cardiovascular diseases, liver-, renal-, and lung-related pathologies, metabolic disease, autoimmune, infections, inflammations, and other neoplastic diseases

**Table:** (b) Healthy subjects

Inclusion criteria	Age	18–45 years
	Sun exposure	UV index 5
	Nutrition, body mass index	Normal, 18.5–24.9
	Calcemia, calciuria, phosphatemia, alkaline phosphatase, parathormone, and C reactive protein.	Normal values

Exclusion criteria	Age	>18 years; <45 years
	Pregnancy	Yes
	Condition present	Drug and/or alcoholic addiction
	Treatment present	Systemic treatment with hormones, antidepressants, antioxidants, and immunomodulators
	Other pathologies	Neurological, physiatrical, digestive, endocrinological, and cardiovascular diseases, liver-, renal-, and lung-related pathologies, metabolic disease, autoimmune, infections, inflammations, and other neoplastic diseases

**Table 2:** Biochemical parameters.

Parameter	Patients	Control	*p* significance (patients versus control)
Lipids (g dL^−1^)	618.7 ± 26.4	601.0 ± 37.1	0.741
Triglycerides (mg dL^−1^)	89.9 ± 18.6	83.4 ± 16.6	0.449
Bilirubin (mg dL^−1^)	0.22 ± 0.03	0.19 ± 0.08	0.811
Calcemia (mg dL^−1^)	8.87 ± 0.30	8.76 ± 0.24	0.902
Calciuria (mg/24 h)	162.6 ± 31.4	153.9 ± 20.7	0.381
Alkaline phosphatase (U L^−1^)	216.4 ± 31.5	209.7 ± 23.1	0.428
iPTH (pg mL^−1^)	27.2 ± 7.4	31.0 ± 8.7	0.517
Phosphatemia (mg dL^−1^)	3.6 ± 0.9	3.4 ± 0.6	0.726
CRP (mg dL^−1^)	0.39 ± 0.23	0.13 ± 0.15	0.121
LDH (U L^−1^)	396 ± 79	203 ± 62	0.014
IL-8 (pg mL^−1^)	68.1 ± 16.3	10.2 ± 4.5	0.001
25(OH)D (ng mL^−1^)	15.9 ± 11.8	26.8 ± 14.4	0.003

CRP: C reactive protein; iPTH: intact parathormone; LDH: lactate-dehydrogenase; IL-8: interleukin-8; and 25(OH)D3: 25-hydroxyvitamin D3.

**Table 3:** Serum 25-OH vitamin D3 concentration in patients and healthy donors.

Vitamin D3	25-OH vitamin D3 (ng mL^−1^)	Patients (number)	Control (number)
Very severe deficiency	1.6–5	2	0
Severe deficiency	5–10	11	2
Deficiency	10–20	44	9
Suboptimal provision	20–30	24	36
Optimal level	30–50	7	38
Upper level	50–70	0	3
Overdose, but not toxic	70–150	0	0
Intoxication	>150	0	0

**Table 4:** Statistical correlations between serum 25-OH vitamin D3, IL-8, and LDH in patients group and controls.

Investigated serum parameters (correlated pair of variables)	Cutaneous melanoma		Controls	
	*r*	*p*	*r*	*p*
25-OH vitamin D3 versus IL-8	−0.650	0.005	−0.143	0.435
25-OH vitamin D3 versus LDH	−0.426	0.021	0.062	0.828
IL-8 versus LDH	0.311	0.006	0.009	0.979

*r*: correlation coefficient; *p*: statistical significance.
